# Evaluation of obstetric outcomes in adolescent pregnancies according to age groups

**DOI:** 10.1590/1806-9282.20250010

**Published:** 2025-08-08

**Authors:** Hakki Serbetci, Atakan Tanacan, Osman Onur Ozkavak, Murat Haksever, Esra Karatas, Mehmet Utku Basarir, Ozgur Kara, Dilek Sahin

**Affiliations:** 1 Turkish Ministry of Health, Ankara City Hospital, Perinatology Clinic – Ankara, Turkey.; 2 Turkish Ministry of Health, University of Health Sciences, Ankara City Hospital, Department of Obstetrics and Gynecology – Ankara, Turkey.

**Keywords:** Adolescent pregnancy, High-risk pregnancy, Refugees

## Abstract

**INTRODUCTION:**

The aim of the present study was to gain insight into how age may influence the outcomes of adolescent pregnancies and to gain a better understanding of the proportion of immigrants in adolescent pregnancies.

**METHODS:**

This retrospective study was conducted in a perinatology clinic between 2021 and 2024. The patients were divided into two categories such as adolescents and adults. The adolescent cohort was then divided into two subgroups according to age. Demographic characteristics, pregnancy follow-up, and postnatal outcomes were reported and compared between groups.

**RESULTS:**

A total of 123 adolescent pregnancies compared with 123 adult pregnancies. There were 27 patients in the 14–15 age group and 96 patients in the 16–17 age group. The adult group exhibited significantly higher age (p<0.01), gravidity (p<0.01), parity (p<0.01), gestational age at birth (p<0.01), neonatal weight (p<0.01), and both antepartum (p=0.017) and postpartum (p<0.01) hemoglobin values compared to the adolescent group. The decline in hemoglobin levels in the peripartum period was significantly higher in the 14–15 age group (p<0.01). A significant difference was observed between adolescent and adult pregnant groups in terms of early pregnancy follow-up. The proportion of immigrants was much higher in the adolescent group at 49.6% and in the adult group at 4.8% (p<0.01).

**CONCLUSION:**

Adolescent pregnancies represent a significant public health concern. The risks in this regard are amplified with a reduction in the age of the patient. The prevalence of adolescent pregnancies among refugees appears to be a matter of significant concern.

## INTRODUCTION

The issue of adolescent pregnancy represents a significant public health concern that necessitates a comprehensive assessment, including the adolescent mother and the associated challenges. Although the incidence of adolescent pregnancies is on the decline globally, they remain a significant public health concern, affecting approximately 25% of women^1,2^. A number of factors have been identified as contributing to the risk of adolescent pregnancy, including low educational level, absence of a partner, lack of self-confidence, alcohol and drug use, inadequate knowledge about sexuality, and inappropriate contraceptive use^3^.

A delay in the diagnosis of pregnancy, lack of prenatal care, and perinatal and neonatal complications are associated with an increased risk of adverse outcomes in this patient population. Adolescent pregnancies are associated with a number of adverse perinatal outcomes, including preterm delivery, intrauterine growth retardation, preeclampsia, gestational diabetes, stillbirth, and intrapartum fetal demise^4,5^. The greatest risk of adverse perinatal outcomes is observed in pregnancies occurring within two years of menarche. The younger the patient, the greater the risk of complications. It has been postulated that the immature pelvic structures of adolescent mothers are associated with an increased risk of shoulder dystocia, intrapartum asphyxia, and stillbirth^6^. In neonates, adolescent pregnancy is associated with an increased risk of low birth weight, respiratory diseases, birth trauma, and infant death^7,8^. Furthermore, hypertensive disorders during pregnancy and postpartum hemorrhage are associated with increased maternal morbidity and mortality^9^.

The number of migrants seeking refuge in our country is on the rise^10^. The sociocultural structure of the population in countries of origin for immigrants is a significant factor influencing the age at which women give birth and the number of children they have^11^. The objective of the present study was to examine the influence of age on the outcomes of adolescent pregnancies and to ascertain the proportion of immigrants in adolescent pregnancies.

## METHODS

The study included data from all pregnant women under the age of 18 who gave birth between June 2021 and June 2024 at Ankara Bilkent City Hospital Perinatology Clinic, which is a tertiary center. Our study was evaluated by the local ethics committee and approved with the number 2/373/2024. Data were obtained by retrospectively reviewing the electronic record system and patient files.

In the initial phase of the study, the patients were classified into two categories such as adolescent and adult. Subsequently, the adolescent cohort was divided into two subgroups, comprising individuals aged 14–15 and 16–17 years, respectively. The frequency-matched control group was randomly selected among pregnancies ≥18 years of maternal age. A series of comparisons were made between the two groups with respect to gravidity, parity, gestational age at delivery, rates of cesarean section, neonatal weight, APGAR scores, and the requirement for neonatal intensive care. In addition, hemoglobin values and hemoglobin decreases in the prenatal and sixth-hour postpartum periods were compared. Furthermore, the rates of aneuploidy screening tests and second-trimester ultrasound scans, as well as the rates of migrant patients within the groups, were also evaluated.

Additionally, a comparison was conducted between the groups with respect to composite adverse obstetric outcomes (CAPO)^12^. The outcomes of interest were preterm delivery, low birth weight (less than 2,500 g), preeclampsia, ablatio placenta, preterm premature rupture of the membranes (PPROM), at least one of the APGAR scores below 7, and neonatal intensive care unit hospitalization.

Statistical analyses were conducted using the Statistical Package for the Social Sciences (SPSS.22, IBM SPSS Statistics for Windows, Version 22.0. Armonk, NY: IBM Corp.). The Shapiro-Wilk test was employed to assess the normality of the data. Given that the data were not normally distributed, median and interquartile ranges were utilized for descriptive purposes. The Mann-Whitney U test was employed for the statistical analysis of nonparametric data. A Pearson chi-square test was performed for the statistical analysis of categorical variables, and percentages were used for definition. The p-values less than 0.05 were deemed statistically significant.

## RESULTS

A total of 246 patients were included in the study. The 14–15 age group comprised 27 patients, while the 16–17 age group consisted of 96 patients. The adult patient group comprised a total of 123 individuals.

The adult group exhibited significantly higher age (p<0.01), gravidity (p<0.01), parity (p<0.01), gestational age at birth (p<0.01), neonatal weight (p<0.01), and both antepartum (p=0.017) and postpartum (p<0.01) hemoglobin values compared to the adolescent group.

There were no significant differences between the subgroups of the adolescent group in terms of gravidity, parity, APGAR scores, NICU admission, and prenatal hemoglobin values. The rate of cesarean delivery was higher in the 14–15 age group compared to the other group (51.9 vs. 34.4%), although this difference was not statistically significant (p=0.099). Significant differences were observed in gestational age at delivery, neonatal weight, and postpartum hemoglobin level between the two age groups. The 14–15 age group exhibited significantly lower gestational age at delivery (p<0.01), neonatal weight (p<0.01), and postpartum hemoglobin level (p=0.02). The decline in hemoglobin levels in the peripartum period was significantly higher in the 14–15 age group (p<0.01).

Preeclampsia and PPROM were significantly higher in the adolescent group, but no significant difference was observed between the adult and adolescent groups in terms of placental abruption (p=0.002, p=0.018, and p=0.69, respectively). There were no significant differences between the subgroups of the adolescent group in terms of PPROM and ablatio placenta. However, preeclampsia was observed significantly higher in the 14–15 age group (p=0.34, p=0.209, p=0.69, and p<0.01, respectively).

A significant difference was observed between adolescent and adult pregnant groups in terms of early pregnancy follow-up. The rate of first-trimester aneuploidy screening and second-trimester anomaly scan was found to be statistically significantly higher in the adult group compared to the adolescent group (p<0.01 and p<0.01, respectively).

The proportion of patients who underwent first-trimester aneuploidy screening tests was 14.8% in the 14–15 age group and 30.2% in the 16–17 age group. The second-trimester anomaly scan was performed in 7.4% of the 14–15 age group and 14.6% of the other group. However, these differences were not statistically significant between the groups (p=0.11 and p=0.32, respectively).

In total, 55.6% of patients in the 14–15 age group and 47.9% of patients in the 16–17 age group were identified as immigrants. No statistically significant difference in immigrant rates was observed between the adolescent groups (p=0.42). The proportion of immigrants was much higher in the adolescent group at 49.6% and in the adult group at 4.8% (p<0.01).

Tables 1, 2, and 3 present a summary of the results of the statistical analyses.

**Table 1:** Comparison of demographic data, neonatal outcomes, and laboratory results between the adolescent and adult groups and the subgroups of the adolescent group.

Variable	Age 14–15 (n=27)	Age 16–17 (n=96)	p-value
Age	15 (0)	17 (1)	<0.01
Gravidity	1 (0)	1 (0)	0.53
Parity	0 (0)	0 (0)	0.5
Gestational age at birth	36 (2)	38 (3)	<0.01
C-section ratio	51.9%	34.4%	0.099
Newborn weight	2,700 (560)	3,070 (476)	<0.01
APGAR 1	7 (1)	7 (1)	0.74
APGAR 5	9 (0)	9 (0)	0.6
Antepartum Hb	11.6 (2.3)	11.5 (1.9)	0.61
Postpartum Hb	9.7 (2.4)	10.6 (1.8)	0.02
Peripartum Hb decrease	2 (1.1)	0.9 (1)	<0.01
**Variable**	**Adolescent (n=123)**	**Adult (n=123)**	
Age	16 (1)	27 (7)	<0.01
Gravidity	1 (0)	2 (2)	<0.01
Parity	0 (0)	1 (2)	<0.01
Gestational age at birth	38 (3)	39 (2)	<0.01
Newborn weight	3,025 (560)	3,277 (475)	<0.01
APGAR 1	7 (1)	8 (1)	0.15
APGAR 5	9 (0)	9 (0)	0.94
Antepartum Hb	11.5 (1.9)	12.2 (1.5)	0.017
Postpartum Hb	10.4 (1.7)	11.4 (1.8)	<0.01
Peripartum Hb decrease	1.1 (1)	0.7 (1.3)	<0.01

**Table 2:** Comparison of the rates of aneuploidy screening tests, second-trimester ultrasound scans, and refugee status between the adolescent and adult groups and the subgroups of the adolescent group.

Variable	Age 14–15 (n=27)	Age 16–17 (n=96)	p-value
Aneuploidy screening tests performed	14.8%	30.2%	0.11
Second-trimester anomaly scan	7.4%	14.6%	0.32
Immigrant	55.6%	47.9%	0.48
**Variable**	**Adolescent (n=123)**	**Adult (n=123)**	
Aneuploidy screening tests performed	26.8%	92.3%	<0.01
Second-trimester anomaly scan	13%	91.3%	<0.01
Immigrant	49.6%	4.8%	<0.01

**Table 3:** A comparative analysis of composite adverse perinatal outcomes between adolescent and adult pregnant women, and the subgroups of the adolescent group.

Variable	Adolescent (n=123)	Adult (n=123)	p-value
Preterm birth	17.9%	4.8%	<0.01
Low birth weight	11.4%	1%	<0.01
Low APGAR score	15.4%	2.9%	<0.01
NICU admission	8.9%	1.9%	0.04
Ablatio placenta	3.3%	1.9%	0.69
Preeclampsia	11.4%	1%	0.002
PPROM	13%	3.8%	0.018
CAPO	34.1%	8.7%	<0.01
**Variable**	**Age 14–15 (n=27)**	**Age 16–17 (n=96)**	
Preterm birth	55.6%	7.3%	<0.01
Low birth weight	29.6%	6.3%	<0.01
Low APGAR score	25.9%	12.5%	0.88
Ablatio placenta	7.4%	2.1%	0.209
Preeclampsia	33.3%	5.2%	<0.01
PPROM	18.5%	11.5%	0.34
NICU admission	7.4%	9.4%	0.75
CAPO	74.1%	22.9%	<0.01

NICU: neonatal ıntensive care unit; PPROM: preterm premature rupture of the membranes; CAPO: composite adverse obstetric outcomes.

## DISCUSSION

The results obtained in the present study suggest that adverse perinatal outcomes such as preterm birth, low birth weight, low APGAR scores, and NICU admission are higher in adolescent pregnancies. In extension, it showed that adverse outcomes other than low APGAR scores and NICU admission increased inversely with age in adolescent pregnancies. In addition, hemoglobin decline in the peripartum period was also observed more in the adolescent group. Furthermore, it was noted that screening tests and examinations, which are crucial in the context of pregnancy, were conducted with lesser frequency in the adolescent cohort.

Adolescent pregnancies are pregnancies with an elevated risk to both the mother and the fetus. This is due to the fact that these patients belong to a group with more risky behaviors and maternal physiological and anatomical immaturity. Adolescent pregnancies represent approximately 11% of all pregnancies worldwide. Nevertheless, the aforementioned rate varies considerably from one region to another^13^. The discrepancy in rates can be attributed to a number of factors, with cultural norms and the absence of uniformity in sexual education curricula across different regions being particularly noteworthy considerations. Moreover, the definition of adolescent pregnancy is different among regions and organizations. Although pregnancies under 18 years were included in the present study, the World Health Organization (WHO) defines the age limit as under 19 years of age^14^. For this reason, the maternal age was much more younger in the present study, and the results should be interpreted in this aspect.

The objective of the current study was to examine the differences in outcomes in adolescent pregnancies between age groups. The findings of our study indicate that the gestational age at delivery was significantly lower in adolescent pregnancies in the younger age group. In addition, the younger age group exhibited a lower neonatal weight. A study comparing the data of 294 adolescent pregnant women with pregnant women over 20 years of age revealed that the incidence of preterm delivery was found to be higher in the adolescent group^3^. In a prospective study comparing the outcomes of adolescent and adult pregnant women, it was reported that the duration of pregnancy was shorter and the neonatal weight was significantly lower in the adolescent group^15^. As in the present study, another study examining the effect of the patient's age on pregnancy outcomes found that the frequency of preterm delivery and low birth weight increased in the younger age group^16^. Our findings are consistent with those reported in the existing literature. It is hypothesized that the preterm delivery and low neonatal weight in the lower age group may be attributed to the fact that these patients may not be fully anatomically and physiologically prepared for pregnancy, may not receive adequate care during pregnancy due to their lower educational level, and may be more susceptible to factors such as smoking, which may affect fetal development. Furthermore, the number of immigrant participants was higher in the adolescent group, and this situation might affect the study outcomes. It has been reported that majority of the immigrants live in low socioeconomic conditions, and this may be an important contributing factor for adverse pregnancy outcomes.

In the present study, there was no difference between the groups in terms of prenatal hemoglobin levels. However, hemoglobin levels at six hours postpartum were found to be lower in the younger age group. Concurrently, the decline in hemoglobin concentrations during labor was also found to be greater among women in the younger age group. In their study, Calle et al. observed that adolescents under the age of 17 years were more likely to experience postpartum hemorrhage than those over the age of 17 years^17^. Another large retrospective cohort study revealed that postpartum hemorrhage was more prevalent in young adolescents^18^. The findings of our study align with those reported in the existing literature on this topic. The higher peripartum blood loss observed in the younger age group may be attributed to inadequate oxytocin release, which may result from incomplete development of the hypothalamo-hypophyseal axis. Additionally, the smaller size of the genital tract in younger women may contribute to an increased incidence of genital tract injury.

Screening tests for aneuploidy and a detailed anatomical examination in the second trimester represent essential components of obstetric care worldwide. In the present study, the rates of antenatal screening tests and second-trimester ultrasonography in adolescent pregnant women were found to be significantly lower than those observed in the general population. Moreover, these rates decline in a linear fashion with the increasing age of the patients. There is a paucity of research on this topic in the adolescent population. However, in a Canadian study, the rate of first-trimester prenatal visits in pregnant women under the age of 20 years was found to be lower than in the older age group^19^. In a separate prospective study, it was observed that the first prenatal visit occurred after 28 weeks in 50% of adolescent pregnancies, while this rate was 6.9% in adult pregnancies^15^. When considered in conjunction with the findings of the present study, it can be posited that teenage pregnancies are a predisposing factor for delayed obstetric care. This may be attributed to the delayed reporting of pregnancies by patients to their environment and health professionals for legal or cultural reasons.

Moreover, in the present study, approximately half of the pregnant women aged 14–17 years were immigrants. The phenomenon of irregular migration represents a significant challenge for societies across the globe. In addition to the cultural differences of the migrant population, inadequate adaptation to the country in which they settle can lead to significant problems in the delivery of health services. It is postulated that immigrant patients may encounter difficulties in accessing healthcare due to circumstances such as undocumented immigration, language, and cultural differences. It is hypothesized that this situation also contributes to a delay in obstetric care.

In our study, the adolescent group exhibited a higher prevalence of composite adverse perinatal outcomes, including preterm delivery and low birth weight. A correlation was observed between a reduction in maternal age during pregnancy and an elevated incidence of low APGAR scores and NICU admissions. The findings of our study are in accordance with those of other similar studies in the existing literature. The aforementioned results represent a synthesis of the findings previously discussed. These findings lend support to the notion that adolescent pregnancies represent a significant public health concern that warrants further investigation on a global scale.

In conclusion, adolescent pregnancies represent a significant public health concern, with adverse obstetric outcomes including preterm delivery, low birth weight, postpartum hemorrhage, and delayed prenatal care. The risks in this regard are amplified with a reduction in the age of the patient. It is vital that physicians and other healthcare professionals are cognizant of the risks associated with adolescent pregnancies and that they provide follow-up care to their patients in a manner that reflects this awareness.

## Data Availability

The datasets generated and/or analyzed during the current study are available from the corresponding author upon reasonable request.
